# Assessing soil erosion and farmers’ decision of reducing erosion for sustainable soil and water conservation in Burji woreda, southern Ethiopia

**DOI:** 10.1038/s41598-024-59076-6

**Published:** 2024-04-15

**Authors:** Shibru Chuda Djillo, Kebede Wolka, Daniel Assefa Tofu

**Affiliations:** 1 Office of Agriculture and Natural Resource, Burji Woreda, Burji, Ethiopia; 2 Wondo Genet College of Forestry and Natural Resource, Hawassa University, Shashemene, Ethiopia; 3 School of Natural Resource Management, Ambo University, Ambo, Ethiopia

**Keywords:** Sustainable use, Crop yield, Slope, Rill erosion, Adoption, Watershed, Environmental sciences, Ecosystem services

## Abstract

Inadequate conservation practice affects the sustainable production of agricultural watersheds due to erosion and fertility decline. Understanding soil erosion and implementing site-specific conservation practice could enhance agriculture-based rural development. The study was aimed to document soil erosion problem and soil and water conservation effort. The specific objectives of this study were to assess soil erosion severity, practices to reduce erosion, and determinants of the decision to reduce erosion. Data were collected by interviewing 198 farm household heads, undertaking four focus group discussions, and assessing rill erosion in 10 farm fields in Morayo and Wacho sub-watersheds of southern Ethiopia. Descriptive statistics and binary logit model were applied to analyze the data. Results indicated that many of the farm households, 63% in Morayo and 83% in the Wacho sub-watershed, perceived moderate to severe soil erosion, which is characterized by big rills and small gullies on the farmlands. Rill densities of 231.4 m ha^−1^ and 84.1 m ha^−1^ in the Morayo and Wacho sub-watersheds were observed, respectively. The estimated annual soil loss due to rills was 61.2 and 23.4 Mg ha^−1^ in the Morayo and Wacho sub-watersheds, respectively. The soil erosion from rills alone exceeds the expected tolerable soil erosion (11 tons ha^−1^ year^−1^). Due to erosion, about 90% of farmers perceived farmland degradation as described by a progressive decline in crop yield. Farmers used to practice traditional techniques to reduce erosion and government introduced conservation measures such as soil and stone bunds. However, many farmers did not use well-promoted conservation measures such as bunds, which could have negative impact on long-term erosion control effort and sustainable implementation of the conservation options. Among the assessed explanatory variables, educational level, farm distance from home, slope of the cultivated land, and frequency of extension contact were significantly affected (p < 0.05) farmers’ sustainable use of conservation measures. Development planners and policy makers are advised to consider site-specific and innovative approaches to implement conservation measures in sustainable approach in the smallholder crop-livestock mixed agriculture system.

## Introduction

Soil erosion by water is a challenge affecting environmental quality in many regions of the world including China^1^, Europe^2^, and North America^3^. Sub-Saharan Africa is also among the regions that are considerably affected by severe soil erosion^4^. About 25 percent of the world’s degraded land is in Africa, and it is estimated that 65% of Africa’s agricultural land is degraded mainly due to soil erosion^5^. In Africa, every year, about 6.5 million ha of cropland is abandoned for different periods due to severe soil erosion^6^, which could have a considerable impact on land productivity and food security.

Soil loss in Ethiopia exceeds the estimated soil formation rate (about 11 Mg ha^−1^ yr^−1^)^4,7^. Even though the severity of soil erosion differs spatially and temporally on cultivated lands following differences in rainfall, soil properties, topography, crop types, and implementation of effective conservation measures, more than 50% of the countrywide soil loss is only from cultivated lands^4,8^. High rates of soil erosion in Ethiopia are mainly caused by extensive removal of vegetation for wood collection, cultivation, and grazing^9,10^. The water flow along land surface in the form of sheet and in small channel named rill are the common forms of water erosion occurring on cultivated lands, which remove a considerable quantity of soil unnoticeably^11^. There are variations in the form and amount of erosion spatially and temporally, and thus site-specific studies are important to undertake informed planning and sustainable management^12,13^.

Soil erosion has been assessed using various techniques depending on resource, temporal and spatial coverage, and skill. Rill erosion, which is one of erosion features mainly occurring on cultivated lands, with depth of up to 30 cm, can be estimated using field survey^14^. Rill erosions are visible features, which farmers could recognize loss of cultivated soils^15^. In addition to the eroding capacity of surface runoff, the flow of water through the channel of rill could easily transport detached soil to downslope. Thus, applying farmers perception as well as field assessment could help understanding of erosion to certain extent. A study estimated varying soil losses (13.5 and 61t ha^−1^) for two watersheds for the same season^16^. Lemma et al.^17^ estimated 3.7 t ha^−1^ soil loss due to rill erosions.

An institutionalized soil and water conservation program focusing on construction of soil and stone bunds, through Ethiopian Ministry of Agriculture, was aimed to reduce soil degradation, improve agricultural production, and enhance food security, and reduce poverty^18–20^. In the past fifteen years, community-based and nationwide soil and water conservation campaigns were widely promoted to support soil and water conservation^21,22^. However, soil and water conservation technologies are not equally successful or effective in different parts of the country. Farmers’ participation in soil conservation activities is influenced by inadequate expert follow-up and assistance, approaches followed in planning and implementations, farmers' landholding size and technical skills^6,23,24^. Lack of effective community participation, limited sense of responsibility over assets created, inefficient implementation of technologies, inadequate policies, lack of integration among stakeholders, unmanageable planning units and evaluation techniques for their feedback affect sustainability^23,25–27^. Studies on factors affecting the use of introduced conservation measures are inconclusive due to temporal and spatial variation in biophysical and socio-economic conditions, suggesting site-specific investigation and understanding.

In Burji woreda, southern Ethiopia, the traditional stone bunds have been constructed for soil and water conservation for centuries. On the other hand, the woreda is one of the areas experiencing severe land degradation resulting from soil erosion. In recent decades, the government implemented some erosion-reducing techniques such as soil bunds and related physical measures such as *Fanny juu*. Despite many years efforts, awareness creation and investment by the government, the existing situation of erosion and impacts of erosion reducing effort have not been well studied. Objectives of this study were (1) to assess perceived soil erosion severity based on farmers’ opinion and rill erosion measurements, (2) to examine farmers effort to implement erosion reducing measures in farmland, and (3) to identify determinants of farmers’ decision to reduce erosion and to manage conservation measures sustainably. The study was conducted in a rarely documented region of southern Ethiopia, Burji woreda, by using household interviews and field measurements of rill erosion in two sub-watersheds.

## Materials and method

### Site description

**Figure 1:**
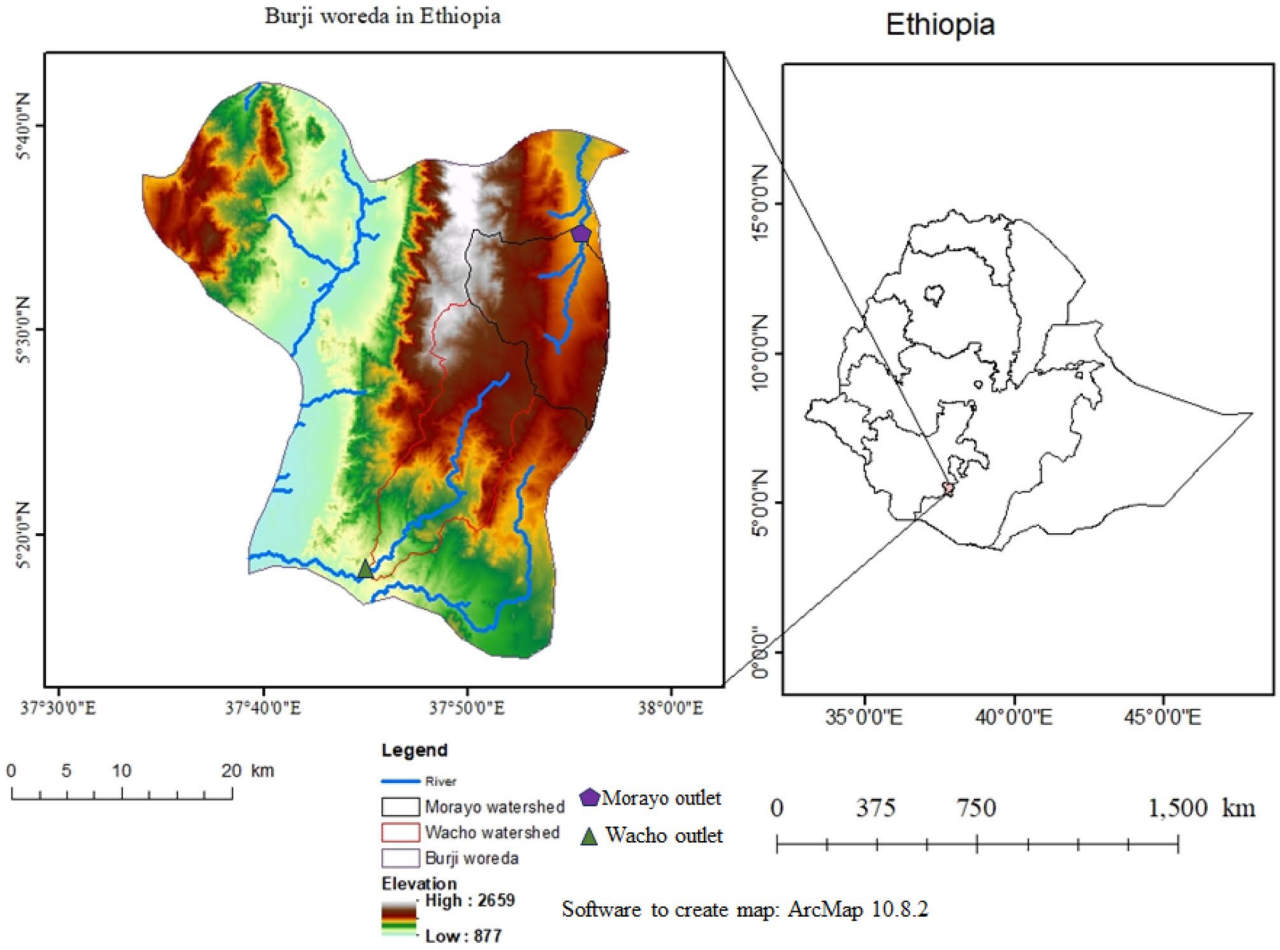
Location of study area (Burji woreda and watersheds) in southern Ethiopia.

The study was conducted in Burji woreda in southern Ethiopia. The woreda is situated between 5° 13′ 18″ N to 5° 42′ 00″ N latitude and 37° 34′ 2″ to 37° 58′ 2″ E longitude (Fig. 1). The mean annual rainfall of the area is 900 mm, with bimodal pattern, where the first rain season is in February–June, locally called ‘Belg’, and the second season is from August–November, which named ‘Meher’. The mean minimum and maximum temperatures of the woreda are 15.1 °C and 27.5 °C, respectively. The undulating relief, dissected plateaus, mountains, and valleys in the elevation of 877–2670 m above sea level characterize the area (Fig. 1). The dominant soil types of the area have been identified as chromic vertisols, dystric fluvisols, dystric nitisols, eutric fluvisols, eutric nitisols, orthic acrisols, and orthic luvisols^28^. Segen and Gelan River watersheds are among the major surface water drainage systems of the woreda.

The main economic activity of the inhabitants of Burji woreda is small-scale subsistence agriculture that relies on growing crops and livestock. The farmers in the area dominantly practice mono-cropping. Majority of the populations live in rural area and rely on subsistence farming as the livelihood strategy, obtaining their livelihood from teff (*Eragrostis tef*), haricot bean (*Phaseolus vulgaris*), coffee, and livestock. Crops such as wheat (*Triticum aestivum*), barley (*Hordeum vulgare*), sorghum (*Sorghum bicolor*), millet (*Panicum miliaceum*), maize (*Zea mays*), bean (*Vicia faba*), and peas (*Pisum sativum*) are also grow in the area. Cattle, goats, sheep, and equines are part of the farming system in the area. Many of the inhabitants depend on rainfall based seasonal farming.

### Methodology

#### Sampling procedure and data collection

The data collection was carried out using multi-stage sampling technique. Burji woreda was selected purposively as it is one of the areas where soil erosion and conservation impacts has not been assessed systematically. From the woreda, two sub-watersheds, viz, Morayo sub-watershed from Gelana River watershed and Wacho sub-watershed from the Segen River watershed were selected because of the existence of diverse agro-ecology (high, mid, and low elevations), and their proximity to each other. Then, from the two sub-watersheds, four kebeles (lowest government administrative unit) were selected. Following the administrative unit is important to select the respondents as they could trustfully respond and cooperate through the administrative system.In Morayo sub-watershed, two kebeles (Bila and Gera) were selected out of the six kebeles. In the Wacho sub-watershed, two kebeles (Gemyo and Kilcho) were selected out of five kebeles. In both sub-watersheds, selections of the kebeles were considered agro-ecological distribution. That means, representativeness to comparatively low, mid and high elevations within sub-watersheds was taken into consideration. This is important as the land use, agricultural practice, and susceptibility to erosion could vary with elevation (agro-ecology). Farmers in the selected kebeles were grouped into wealth categories (rich, medium, and poor) based on local criteria (size of farm area, number of livestock, status of the house, productivity of the farmland, overall income). Farmers possessing > 2 ha productive farmland, above 10 cattle, and house built of more than 70 corrugated iron sheets are grouped as rich. Farmers with < 1 ha farmland, < 3 cattle, house with grass roof or less than 30 corrugated iron sheets are considered poor. The economically medium farmers own resources (land, cattle, house etc.), which is greater than poor but less than rich. From the list of farm households in the wealth categories, 198 households were selected randomly using lottery method (Table 1). Household heads were responded on socio-economic issues, challenges and management of agricultural land, perceived erosion, and methods practiced on controlling erosion, soil and stone bunds management, and challenges in using bunds. The questionnaire contains both open-ended and close ended questions depending on the context of the question.

**Table 1:** Sample distributions among the sub-watersheds.

No.	Name of kebeles	No of total household			No of sampled household		
		Male	Female	Total	Male	Female	Total
1.	Bila, Morayo sub-watershed	97	11	108	48	6	54
2.	Gera, Morayo sub-watershed	77	7	84	39	3	42
3.	Gemyo, Wacho sub-watershed	85	6	91	43	3	46
4.	Killcho, Wacho sub-watershed	98	15	113	49	7	56
	Total	357	39	396	179	19	198

Besides the interview of the head of the farm household, one focus group discussion was carried out in each kebele by selecting 12 participants. Participants from different villages within each kebele were considered. Agricultural development experts and kebele administrator assisted in the selection of model and knowledgeable farmers from different villages who participated in the focus group discussion. Farmers’ opinions on erosion and conservation measures, perception towards erosion level and control practice, and functions and management of introduced measures were major issues discussed in the focus group.

#### Rill erosion survey

In each of the two sub-watersheds, 5 representative cultivated fields were selected for the rill erosion survey. In the Morayo, the selected fields were on a slope of 15–38%, and in the Wacho, the fields were on a slope of 8–12%. Based on their relative topographic positions, the surveyed fields were classified into three categories: upslope, mid-slope, and downslope fields. A greater number of mid-slopes (three out of five fields) were selected because many of the farm fields are in this topography. The length, width, and depth of rills in each field were measured manually with measuring tape and ruler. The width of a rill varied across its depth and length. Depending on the depths and lengths, therefore, widths were measured at many points along the length. Likewise, depth measurements were taken at many points along the length^17^. In cases of rills that came laterally and merged with a main rill, the length was measured from its starting point to the confluence with another rill. For bulk density estimation, core samples of topsoil (0–10 cm) were taken from each field and after oven drying and weighing, the bulk density of the known volume of soil was computed.

To determine the eroded soil volume, each rill was divided into homogenous segments, for which length, average depth, and average width were determined. The product of the depth, width, and length parameters gave the rill volumes, which is equivalent to the volume of soil lost due to the rills. The total volume of soil lost was obtained by summing the estimate for each rill segments. The eroded soil volume was also expressed in terms of the weight of the eroded soil by multiplying the calculated volume by the measured bulk density. The area of the actual damage, the surface area covered by the rills themselves, can be estimated from the product of the length and width of each rill. Rill density, ratio of total rill length to the farm area, in each farm was estimated. It was recognized that the estimated soil loss is only the best approximations of erosion due to the rills, and it excludes soil loss by the inter-rill erosion processes. The measurement was carried out in one cropping season. The length and width of rills could be dynamics with rainfall severity and land management, which were not considered in this study.

### Data analysis

The data were analyzed by applying descriptive statistics and econometric models in Statistical Package for Social Science (SPSS) version 20 computer software. Descriptive statistics such as mean, and percentage were used to summarize farmers’ socio-economic characteristics and perception of erosion and soil and water conservation. The result for rill erosion measurements were summarized and presented descriptively. A binary logistic regression model was used to assess the relationship between multiple independent variables and categorical dependent variables (continuously use SWC practices or not). The independent variables considered in the binary logistic regression model were farmers age, sex of head of the household, level of formal education, family size, farm size, the slope of the farmland, livestock holding, contact with extension service providers, perceived labor availability, the distance of farm from road and residence, perception on erosion severity, perceived land ownership security, and participation on soil and water conservation training. Those independent variables are assumed to affect attention and interest of farmers to control erosion. Other studies also considered many of them in estimating determinants of physical SWC^15,29,30^.

### Ethics approval and consent to participate

Ethical clearance was obtained from the Research Ethical Committee of Wondo Genet College of Forestry and Natural Resource, Hawassa University, and permission and a supporting letter were obtained from the Burji woreda office of Agriculture before data collection. Verbal informed consent from each participant was obtained during data collection. The respondents were given the right to respond or refuse on the questionnaire for the study. During data collection confidentiality was assured for all participants, farmers, and experts. Relevant ethics, guidelines, and regulations were followed when using the methods for data collection.

## Results and discussion

### Socio-economic characteristics of household

In the Morayo and Wacho sub-watersheds, the mean age of household heads who decided to use SWC measure was about 42 years. The mean non-users age was about 47 and 37 years in Morayo and Wacho sub-watersheds, respectively, (Table 2). This implies that the heads of household, both non-users and users of SWC measure, are active for labor work required in agricultural activities. In addition, many of the respondents were male as head of the household, who should be male traditionally, was allowed to respond to the questionnaire. The mean family number between the users and non-users of the SWC structure was not differing much in both sub-watersheds, implying other factors are more important in managing the SWC measure. In the Morayo and Wacho sub-watersheds, many of the non-users of SWC structures are households led by illiterate people. This would highlight the importance of formal education to accept and implement agricultural technologies such as SWC measures, which require information on technical aspect and interest to longer planning horizon. The educated person could explore different technologies and options through local and international media and exert effort to better production and land management^31^.

**Table 2:** Age, sex, family size and education status of soil and water conservation structure user and non-user farmers in the Marayo and Wacho sub-watersheds, Burji woreda, southern Ethiopia.

	SWC structure user (%)		Non-user of SWC structure (%)		Total (%)	
	Morayo, n = 66	Wacho, n = 90	Morayo, n = 30	Wacho, n = 12	Morayo, n = 96	Wacho, n = 102
Age of household head						
15–29	19.7	21.1	20.0	0.0	19.8	18.6
30–44	54.6	52.2	40.0	75.0	50.0	54.9
45–64	24.2	26.7	36.7	8.3	28.1	24.5
> 65	1.5	0.0	3.3	16.7	2.1	2.0
Mean age, year	41.55	42.3	46.7	36.7	43.15	41.7
Sex of the respondent						
Male	87.9	92.2	93.3	75.0	89.6	90.2
Female	12.1	7.8	6.7	25.0	10.4	9.8
Family size						
1–5	15.2	12.2	13.3	16.6	14.6	12.7
6–10	71.2	47.8	63.4	41.7	68.8	47.1
11–15	13.6	32.2	13.3	41.7	13.5	33.3
> 15	0.0	7.8	10.0	0.0	3.1	6.9
Mean, number	7.2	9.28	8.4	8.16	7.58	9.15
Education level						
Illiterate	0.0	8.9	66.7	66.7	20.8	15.7
Reading and writing	57.6	52.2	30.0	33.3	49.0	50.0
Primary school	36.4	28.9	3.3	0.0	26.0	25.5
Secondary school	6.0	10.0	0.0	0.0	4.2	8.8

In Morayo sub-watershed, the average landholding of the households that decide to use and not to use SWC structure was 1.29 and 1.01 ha, respectively (Table 3). In Wacho sub-watershed, the average land holding was 2.58 ha for SWC users and 0.66 ha for non-users of SWC structures (Table 2). In both sub-watersheds, the non-users of SWC measures possess comparatively smaller land holdings than users of the same measures. The t-test comparison showed that the mean landholding size difference between the two groups (user and non-user) was found to be significant (p < 0.05). This could be perhaps associated with the nature of the SWC structures that occupy the cultivable areas, due to which households possessing smaller land areas are less motivated to practice the measure. A household with greater cultivated land has the option to avert risk of land wasting due to SWC structures.

**Table 3:** Land size, ownership, slope and distance from living area of soil and water conservation structure user and non-user farmers in Marayo and Wacho sub-watersheds, Burji woreda, southern Ethiopia.

	SWC structure user (%)		SWC non-user, of %		Total, %	
	Morayo, n = 66	Wacho, n = 90	Morayo, n = 30	Wacho, n = 12	Morayo, n = 96	Wacho, n = 102
Land size, ha						
0.25–0.5	12.1	5.6	20.0	38.3	14.6	11.8
0.51–1.00	48.5	4.4	50.0	25.0	49.0	6.9
1.01–1.5	19.7	16.7	20.0	16,7	19.8	16.7
1.51–2	7.6	23.3	10.0	0.0	8.3	20.5
> 2	12.1	50.0	0.0	0.0	8.3	44.1
Mean (ha)	1.29	2.58	1.01	0.66	1.2	1.9
Land ownership						
Owned	98.5	93.3	80.0	25.0	92.7	85.3
Rented	1.5	6.7	20.0	75.0	7.3	14.7
Slope gradient						
Flat	18.2	8.9	36.7	100	24.0	19.6
Moderate	45.5	24.4	56.7	0.0	49.0	21.6
Steep	36.3	66.7	6.6	0.0	27.0	58.8
Farmland distance, minute						
< 30	15.2	23.3	20.0	8.3	16.7	21.6
31–60	65.1	67.8	43.3	25.0	58.3	62.8
61–90, %	19.7	8.9	36.7	41.7	25.0	12.7
91–120, %	0.0	0.0	0.0	2.5	0.0	2.9

In the Morayo sub-watershed, 98.5% of the respondents have their own land for cultivation and 80% of non-users of SWC structures have their own land. In the Wacho sub-watershed, 93.3% of the users of SWC structures and only 25% of the non-users have their own land. Because farmers who have rented land have no long-term planning horizon to invest in that land since the maximum period of renting is not more than two or three years, but the benefit gained from conservation structure is not as such immediate^32^. Restoration of soil fertility after constructing SWC structures and the impact of erosion in the fields lacking conservation structure take some years to realize^33^.

Slope is one of the physical characteristics of farm plots used as a factor for erosion, which in tum affects the use of SWC measures. The response of farmers about soil conservation structures showed differences among farmers cultivating different slope categories. Many of the households not using physical SWC measures own land with flat or moderate slope, but those who use SWC structures mainly own moderate or steep sloping land (Table 3). The steep slope could aggravate erosion and thus, farmers tend to apply conservation measures. The conventional cultivation, which often plows the land three times before sowing expose the soil for erosion particularly on sloping lands.

The average number of 3.7 oxen, 2.2 cows, 1.4 donkeys, 2.2 goats, 0.3 sheep, and 0.4 calves is owned by the farmers with an average tropical livestock unit (TLU) of 10.27. Livestock is an important component of the farming system for generating income, draft power, and food (milk, meat). In the Wacho and Morayo sub-watersheds, livestock challenges the sustainability of conservation measures that have been constructed in the cultivated field. The farmers and key informants underlined that livestock interference due to free grazing practices in the area is a major limitation to sustainable management of SWC structures. Especially after crop harvesting season, open and free livestock grazing on the field destroys conservation measures. Open grazing on cultivated land after crop harvest is a challenge in Kenya and different regions of Ethiopians^34^. On the contrary, traditional farming practices in some parts of the county, for instance, in Konso, where livestock are stall-fed, conservation practices are free from livestock pressure and as a result, structures become part of the sustainable landscape management practice^35^. In the absence of the willingness of farmers to take conservation as part of farming activities and the low commitment of government and NGOs to introduce improved livestock husbandry and management policies, conservation interventions would never be sustained. The open grazing of livestock causes of the destruction of SWC structures and vegetation.

In Morayo sub-watershed, 71.2% of users of SWC structure farmers and 90% of the non-users, have access to the communal grazing lands to feed livestock. In Wacho sub-watershed, 80% of the users and almost all non-users benefit from grazing on communal lands. Even though this could reduce trampling and residue grabbing on cultivated lands, it would result in overgrazing and land degradation due to little conservation effort to common resources^36^.

In less educated rural areas, extension services could support the learning processes of agricultural technologies of land, livestock, and crop management. Agricultural extensionists were assigned in the kebele and expected that all farmers in the study area have access to the extension services. Our study showed that majority of the non-users of SWC structures in the Wacho sub-watershed could visit the experts every 15 days, but those in Morayo sub-watershed visit every three months. In both sub-watersheds, majority of users of SWC structures visit the extension agent 1–3 times per month. In addition, majority of the users of SWC have received technical training on SWC structures, but non-users mainly in Wacho sub-watershed did not get training. The woreda and kebele might have principles on extension service and training. Resource could be limiting factor to give sufficient training for all farmers. In any reason, less access to extensionists and training could reduce the chance to practice SWC structures as these are related to the opportunity to acquire skill and knowledge.

### Soil erosion

#### Farmers' perception of soil erosion

Farmers' perception of the severity of erosion and its impacts on their agricultural products could affect implementation and management of conservation measures. A greater proportion of the farmers, 63% in Morayo and 83% in the Wacho sub-watershed, perceived moderate to severe soil erosion. The survey results revealed that farmers in Wacho sub-watershed faced more severe erosion problems than farmers in the Morayo sub-watershed. Among farmers who perceived severe soil erosion in the whole study area, 59% were from a steep slope dominating area, Wacho sub-watershed. About 36% of the farmers in the Morayo sub-watershed and 15% in Wacho sub-watershed observed minor erosion features on their cultivated lands. Farmers associate severity of soil erosion mainly with land surface dissection such as gullies and big rills but may ignore the sheet and minor rills even though those could indicate the removal of soil^37^. In the Wacho and Morayo sub-watersheds, about 90% of farmers perceived the problem of land degradation on their farm plots as characterized by a progressive decline of crop yield. Even though extent of nutrient replenishment affects cropland productivity, decline of crop yield could also be associated with gradual decline of nutrient by erosion from surface soil.

**Figure 2:**
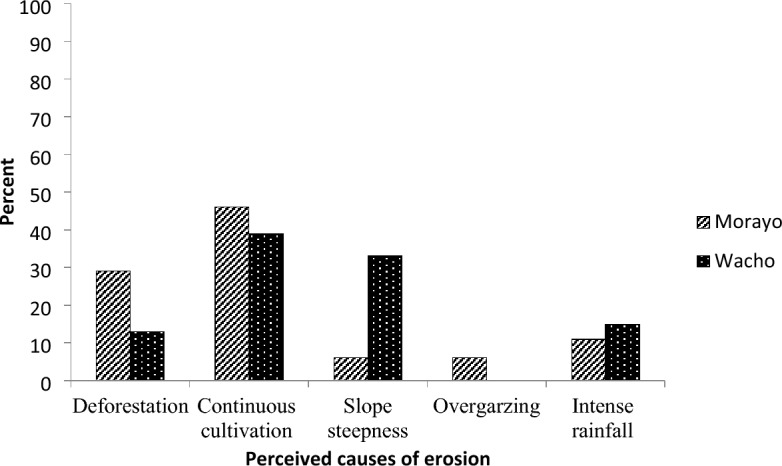
Responses of households on the causes of erosion in Marayo and Wacho sub-watersheds, Burji woreda, southern Ethiopia.

Across sub-watersheds, farm households have different perceptions regarding the causes of soil erosion and crop yield-related land degradation. In the Morayo sub-watershed, farmers perceived that continuous cultivation (46%), deforestation, (29%), excess rainfall (11%), slope steepness (6%), and overgrazing (6%) were major causes of erosion. In the Wacho sub-watershed, continuous cultivation (39%), slope steepness (33%), excess rainfall (15%), and deforestation, (11%) were perceived as some of the major causes of soil erosion problems (Fig. 2). In practice, a given management practice or factor would have a greater contribution to degradation and erosion, but the resultant impact is often due to a combination of more than one factor and practice.

#### Rill erosions in the selected farms

In all 10 cultivated fields where rill assessment conducted, rill erosions were observed. A total of 121 and 228 rills were observed in the Morayo and Wacho sub-watersheds, respectively. The total lengths of the assessed rills represented rill densities of 231.4 m ha^−1^ in the Morayo sub-watershed, and 84.1 m ha^−1^ in the Wacho sub-watershed. The total volume of all the rills was ∼ 51 m^3^ ha^−1^ in the Morayo. This is equivalent to 61.2 Mg ha^−1^ of soil loss in the respective time given the average measured soil bulk density is 1.2 Mg m^−3^. In the Wacho, the total volume of all the rills was ∼ 18 m^3^ ha^−1^. This is equivalent to 23.4 Mg ha^−1^ of soil loss in the respective time given the soil bulk density of 1.3 Mg m^−3^.

Because of the exclusion of inter-rill and sheet erosions, the estimated erosion rates would be an underestimate of the actual rate of soil loss. Compared to the average soil loss rates estimated to occur from cultivated fields in the different parts of the country (e.g., 30 Mg ha^−1^ per year^12^; 45 Mg ha^−1^ per cropping season^33^), the estimated soil loss rate in the Wacho was less, whereas the cultivated lands in the Mrayo watershed had greater soil loss. In both sub-watersheds, the soil loss due to rill erosion is greater than the loss estimated by Lemma et al.^17^, which was 3.17 Mg ha^−1^ in northern Ethiopia and 13.5 Mg ha^−1^ in the Kechemo^16^. The rill erosion in the Morayo is as severe as the amount estimated for Erene areas^16^. The severity of soil erosion could be associated with differences in erosion factors including soil properties, topography, and crop management. In the cultivated lands of both sub-watersheds, the estimated soil loss from cultivated fields exceeds the annual soil formation rate of 11 Mg ha^−1^ year^−1^^33^. Rill erosion accounts about 30% of total soil^16^, implying severe soil erosion in the study sub- watersheds. Concurring with the farmers’ perception of severe soil erosion in the area, the estimated high soil loss in the form of rill erosion itself needs sufficient remedial measures for sustainable production.

### Effect of soil erosion and implementation of physical soil and water conservation practices

In the study area, farmers indicated negative impacts of soil erosion. About 50% of the users of physical SWC and 33% of non-users in the Morayo sub-watershed perceived soil depth decline due to erosion. In the Wacho sub-watershed, about 30% of the physical SWC users and non-users perceived the negative effect of erosion on the soil depth. Supporting farmers' perception, Wolka et al.^33^ reported annual soil depth loss of about 4 mm on farm plots with an erosion rate of about 50 t ha^−1^ year^−1^. Practically, the erosion taking place on the soil surface could reduce the depth of fertile topsoil, particularly in upper slopes, and may deposit on flat lands. The loss of fertile topsoil could reduce the functioning of soil including water-holding capacity and fertility for crop production. While from non-users of SWC practice, 66.7% of the household heads perceived that erosion reduces yield and yield components. About 49% and 41% of users of physical SWC measures in the Morayo and Wacho sub-watersheds, respectively, and 68% and 75% of non-users of physical SWC measures in the Morayo and Wacho sub-watersheds, respectively, perceived crop yield declines due to erosion. The loss of fertile surface soil by water erosion could reduce the soil quality and thus can affect crop yield.

Traditional cutoff drain, a small structure constructed on cultivated fields using oxen-plow, is the widely used practice by farmers in Morayo sub-watershed (46%), practiced for protecting the soil, nutrient, and seeds from erosion. It is also constructed on flat farmlands to protect against water logging problems. The low labor requirement and temporal erosion-protecting advantages of traditional cutoff drains were explained during the focus group discussions. Such traditional practice has also been practiced in the other area in southern Ethiopia^15^.

Through government initiatives and public campaigns, different SWC structures such as bunds have been introduced, promoted, and implemented in the study area. In the Morayo sub-watershed, 90.9% of the SWC structure users constructed soil bunds on their farmland and 46.7% of non-adapters have a positive view of soil bunds but do not use them on their farm. In the Wacho sub-watershed, only 24.4% of the SWC structure user farmers applied soil bunds on their farmland (Table 4). The percentage of farmers implementing soil bunds is greater in Morayo as soil bunds require less labor because the excavated material from the ditch is thrown downhill^33^. The availability of stones near the farmland affects the preference for stone bunds. About 96% of the users of physical SWC constructed stone bunds in the Wacho sub-watershed because of the availability of stones, but only 12% of users in the Morayo sub-watershed implemented stone bunds. *Fanya juu* terraces, a type of SWC practice made by digging a ditch and throwing the soil uphill (opposite to soil bunds), are implemented by 73% of user farmers in the Morayo and only 4% in the Wacho sub-watershed. During the discussion with key informants in the Wacho sub-watershed, farmers explained that they have little knowledge and interest in *Fanya juu* terraces. The disadvantage of this structure, as explained by the farmers, is that it creates a water logging at its upslope, and the embankment is washed easily when rainfall is high. Practically, this could occur as the embankment of *Fanya juu* impound water on cultivable plot but in soil bund the runoff is collected in the channel.

**Table 4:** Perceived effects SWC structures in Marayo and Wacho sub-watersheds, Burji woreda, southern Ethiopia.

Attitudes on effect of SWC	SWC user (%)		Non user of SWC (%)	
	Morayo N = 66	Wacho n = 90	Morayo n = 30	Wacho n = 12
Improve crop production	34.9	45.6	43.3	58.3
Reduce soil loss	37.9	25.6	30	33.4
Improve soil fertility	9	17.8	20	8.3
Reduce surface runoff	18.2	11	6.7	0

In both Morayo and Wacho sub-watersheds, both users and non-users of SWC structures perceived positive roles of physical SWC, including improving crop production, reducing soil loss, improving soil fertility, and reducing surface runoff. Farmers also explained that the removal of stones for the construction of stone bunds, especially in the lower and middle parts of the sub-watershed, made the plot better for farming. The positive opinion on those conservation structures could encourage at least some farmers in construction and management.

### Factors affecting sustainability of SWC structures

The binary logistic regression model was used to identify determinant factors affecting the farmers’ use of introduced soil and water conservation structures. Among the assessed explanatory variables educational level, farm distance from home, the slope of the cultivated land, and frequency of extension contact were significantly affected (p < 0.05) the farmers’ use of soil and water conservation structures. Variables such as age of the household, sex, family size, farm size, perception of land degradation, SWC training, livestock holding, and perceived land security did not show significant (p > 0.05) relation to the use of the SWC structure in the study area (Table 5).

**Table 5:** Factors affecting farmers’ decision to use introduced SWC structures in the Burji woreda, southern Ethiopia.

Variables	B	S.E	Wald	Sig	Exp(B)
Age	− 1.678	0.962	3.042	0.081	0.187
Sex	0.085	2.016	0.002	0.967	1.088
Family size	− 1.041	0.847	1.510	0.219	0.353
Education	6.832	1.925	12.601	0.0	926.81
Livestock	1.108	0.703	2.486	0.115	3.028
Farm size	0.22	0.792	0.077	0.781	1.247
Farm distance	− 1.653	0.809	4.176	0.041	0.191
Slope	3.934	1.251	9.882	0.002	51.097
Land security	− 5.390	13.57	0.158	0.691	0.005
Extension service	1.180	.594	3.951	0.047	3.255
Training	0.226	2.416	0.009	0.926	1.253
Perceived degradation	− 8.092	4.34	3.477	0.062	0.0
Constant	0.587	14.77	0.002	0.968	1.798

As expected, the formal education level of the household head was positively related to the use of improved SWC structures. Educated farmers are presumed to have exposure to new technologies and innovations and are more receptive to new ideas and more willing to use them. This could be associated with access to different information including media, better ability to analyze information, and willingness to development. Concurring with the finding in the study area, a positive effect of formal education on the adoption of SWC structures was reported in the Wollo areas of northern Ethiopia^30^. The slope of the land was associated and related with the use of conservation structures positively and significantly (p < 0.05). Keeping other variables constant, a unit increase in the slope of land from flat to very steep, the probability of farmers using SWC structures would increase by a factor of 51.097, i.e., the odds of farmers that cultivated sloping lands are 51.097 times more likely to use SWC structures than the odds of farmers who cultivating flat lands. This implies that farmers cultivating erosion-vulnerable sloping fields tend to use SWC structures more than farmers that cultivate lower-sloping fields. Farmers take SWC structures more seriously when they are cultivating steep land, for instance, farmers in the Konso area started a unique SWC system because their landscape is dominated by steep slopes^35^. Therefore, the slope has a significant influence on the farmers' decision to construct conservation structures on their cultivated fields. This is consistent with other studies undertaken in other areas^38–40^.

Extension service plays a great responsibility in awareness about SWC practices and the possibility of a farmer using SWC structures. As the frequency of contact with extension service provider increases, the possibility of the farmers using SWC structure increases. Extensionist contact had a positive significant influence on farmer’s decision to practice SWC structure and it increases the adoption level by a factor of 3.26. That is, the odds of farmers that have extension contact are 3.26 times more likely to use SWC structures than the odds of farmers who have limited extensionist contact. Earlier studies reported that access to extension services affected the use of SWC practices positively and significantly^41–44^. Well-informed and trained farmers are more likely to take rational decisions and have longer planning horizons including the construction of SWC structures.

Farm distance from home was hypothesized to be negative on the SWC practices and it decreases the use of SWC structures by the factor of 0.191, i.e., the odds of farmers that have long distances showed 0.191 times less likely to use structures than the odds of farmers that have a short distance from the home. If the distance between the household's home is far, farmers could have less interest to manage their land. Asfaw and Neka^30^ and Belete^45^ reported that limited numbers of farmers frequently inspected and maintain their land whilst the distance increased. Less time and energy are needed to manage closed farmlands than far from their homes as a result they are discouraged from constructing conservation structures on such farmland.

The age of the household head was insignificantly but negatively correlated with the farmers' use of conservation structures, i.e., if the age of farmers increases by one year the possibility to use SWC structures decreases by 0.187. Thus, younger farmers are more likely to use conservation structures than older farmers. This means the decision to the adoption of technologies by older farmers on SWC decreases. Younger farmers have more willingness to adopt SWC structures as they seek knowledge from different sources, and they had long-term plans to undertake SWC practices. This result agrees with other studies^30,44,46^.

The sex of household heads was found to be insignificant but positively related to farmers' decision of constructing SWC structures in the study area. This implies that other things remaining the same, male-headed households appeared more likely to use SWC structures than female-headed households. Also, other studies reported a significant positive relationship between the sex of the household and their decision to use SWC structures^41,44,45,47^. In general, male-headed households have better access to farmland, labor, and agricultural technologies than female-headed households.

The coefficient for family size has a negative sign highlighting that the households with smaller sizes seem to decide to use SWC structures than households with larger family numbers. This is in line with the previous studies by Aklilu^39^, who reported that larger families are less likely to continue using stone bunds. On contrary, researchers reported that farmers who have a greater family are more likely to invest in SWC structures^23,43,45–48^.

## Conclusions

This study aimed to assess soil erosion and farmers’ decision on reducing erosion. The result showed that despite the previous efforts to introduce different conservation measures, soil erosion is a problem affecting agricultural land productivity. Majority of the households, 63% in Morayo and 83% in the Wacho sub-watershed perceived moderate to severe soil erosion. Since farmers recognize the severity of soil erosion based on visible channels such as rills, the severity could be beyond the perceived as there could be considerable unnoticed soil loss. The soil losses in the form of rill erosion alone (61.2 Mg ha^−1^ in Morayo; and 23.4 Mg ha^−1^ in Wacho) exceed the estimated average soil formation rate (11 Mg ha^−1^ year^−1^), highlighting the challenge for sustainable crop production. Farmers use the traditional cutoff of drains to dispose excess surface and thus minimize the effect of erosion on cultivated lands. Farmers perceived that the government introduced SWC structures such as soil and stone bunds are protecting the cultivated land from erosion and nutrient depletion. Farmers are also interested in soil management practices as the structures help improve the productivity of their farmlands. However, many farmers are not using the introduced bunds in cultivated lands, which could have negative implication on the efforts of reducing erosion and long-term soil fertility. Understanding the determinant factors that affect farmers’ decision to use the SWC structures would contribute to the design of appropriate approach in the conservation effort. Among the assessed explanatory variables, educational level, farm distance from home, the slope of the cultivated land, and frequency of extension contact were significantly affected (p < 0.05) farmers’ use of SWC structures. Development workers are advised to consider site-specific innovative approaches to implement and sustain soil and water management techniques in the farming activities. The local extensionist should improve access to training and extension services by considering farmers in the remote areas.

## Data Availability

The datasets are available from the first author on reasonable request.

## Abbreviation

SWC: Soil and water conservation
